# Macrophyte communities as bioindicator of stormwater pollution in rivers: a quantitative analysis

**DOI:** 10.7717/peerj.15248

**Published:** 2023-06-01

**Authors:** Roman Babko, Tetiana Diachenko, Jacek Zaburko, Yaroslav Danko, Tatiana Kuzmina, Joanna Szulżyk-Cieplak, Joanna Czarnota, Grzegorz Łagód

**Affiliations:** 1 Department of Invertebrate Fauna and Systematics, Schmalhausen Institute of Zoology, National Academy of Sciences of Ukraine, Kyiv, Ukraine; 2 Department of Ichthyology and Hydrobiology of River Systems, Institute of Hydrobiology, National Academy of Sciences of Ukraine, Kyiv, Ukraine; 3 Fundamentals of Technology Faculty, Lublin University of Technology, Lublin, Poland; 4 Faculty of Natural Sciences and Geography, Sumy State Pedagogical University, Sumy, Ukraine; 5 Department of Applied Ecology, Sumy State University, Sumy, Ukraine; 6 Department of Environmental Engineering and Chemistry, Rzeszow University of Technology, Rzeszów, Poland; 7 Faculty of Environmental Engineering, Lublin University of Technology, Lublin, Poland

**Keywords:** Macrophytes, Rivers, Stormwater systems, Pollution, Plants communities

## Abstract

Macrophytes are one of the important indicators used in assessing the anthropic impact on aquatic ecosystems. The structure of macrophyte communities of two rivers were compared by species composition, dominant species and projective cover using statistical methods. It is shown that the influence of storm runoff on these rivers is manifested in the form of a change in the dominant species composition. Based on the statistical analysis carried out, it can be argued that, despite the peculiarities of the flora composition of each of the rivers, the influence of storm runoffs largely neutralizes this specificity, determining the situation in local areas immediately below the runoff. In the area of the effluent discharge the dominance of individual species and an increase in the area overgrown with macrophytes was observed. In the area of stormwater discharge on the Psel River, species were usually present: *Nuphar lutea*, *Ceratophyllum demersum*, *Myriophyllum spicatum* and on the Bystrica River—*Glyceria maxima*, *Sagitaria sagittiformis*, *Stuckenia pectinata* and *Potamogeton crispus*. The use of the NMDS method has been found to provide good insight into the structural rearrangements in macrophyte communities affected by runoff from stormwater systems.

## Introduction

Modern inland water bodies—rivers and lakes—have undergone significant changes over the past 100 years, and their ecosystems have largely lost their natural identity. This was mainly due to the entry of human waste (Grant et al., 2012; Aristi et al., 2015) into the hydroecosystems.

There are a number of methods for assessing the negative impacts on water bodies. Traditionally, these methods have been based on assessing the water quality through chemical analyses (Rueda et al., 2002; Gücker, Brauns & Pusch, 2006). However, the quality of natural waters is determined by the physiological activity of organisms, which makes them good indicators (Friberg et al., 2011). As a consequence, the biological approaches to assessing the water quality (Directive 2000/60/EC, 2000) have recently begun to prevail.

To date, various systems for assessing water quality have been developed based on indicator species or communities (Ops etal , 1973; Butterworth, Gunatilaka & Gonsebatt, 2001). It is known that the spectrum of indicator organisms is quite wide, from protozoa to fish. At the same time, researchers are largely focused on the use of organisms, the study of which is quite simple. Higher aquatic plants are the most studied component of aquatic ecosystems. They allow one to quickly and visually assess the ecological state of the reservoir. However, it should be noted that many species of aquatic plants have a wide ecological range of tolerance and their presence is often determined by biological and random factors, and not just by simple environmental determinism.

Aquatic plants are actively studied and used to assess the state of water bodies (Melzer, 1999; Stelzer, Schneider & Melzer, 2005), *e.g.*, they are widely used to classify lakes (Rørslett, 1991; Vestergaard & Sand-Jensen, 2000). Obviously, when assessing the negative impact on flowing water bodies, a number of problems arise related to the effect of current, duration of exposure, displacement in space of the effect from the pollution source, *etc*. Most rivers in Europe have been disturbed by a human: hydrological regime, channel morphology, and floodplain landscapes have been changed (Schmutz & Sendzimir, 2018). The consequence of such disturbances is deterioration in water quality, a decrease in the overall biological diversity and a the ability of hydroecosystems to self-purify (Schmutz & Sendzimir, 2018). A decrease in the water quality in rivers is also caused by point sources of allochthonous pollution, which have a local impact (Madoni & Zangrossi, 2005; Sutton et al., 2011; Grant et al., 2012). The assessment of such sources of water pollution is a separate issue (Merseburger, Marti & Sabater, 2005; Babko et al., 2016; Pliashechnyk et al., 2018).

Among the allochthonous sources of river pollution, the runoff from stormwater systems is relevant (Marsalek et al., 1999; Rossi et al., 2013). Stormwater systems are an indispensable element of urban agglomerations. However, unlike the effluent from enterprises, the volume and composition of which is predictable, these parameters of stormwater are much more difficult to anticipate. Thus, storm runoffs depend on the precipitation regime, and their composition depends on the type of activity in the territories in which they are formed. At the same time, storm runoff can have a very significant impact on rivers, especially small ones (Barałkiewicz et al., 2014; Gołdyn et al., 2018). The impact of storm runoff obviously requires special attention and assessment of the degree of its negative impact on water bodies. In this respect, aquatic plants, the location of which is constant in space (with rare exceptions), constitute convenient means for assessing the quality of the aquatic environment. The simplest approach is to establish the species composition of plants (qualitative approach). These taxonomic or semi-quantitative estimates of the abundance of macrophytes with different ecological characteristics are used most often (Egertson, Kopaska & Downing, 2004; Coops et al., 2007). However, the use of quantitative indicators, one of which is the percentage of coverage of the area of the reservoir, is promising. This indicator is more clearly defined and objective, although it was less frequently included as a standard in monitoring programs (Cheruvelil & Soranno, 2008).

On the basis of the data on the species composition and quantitative development of aquatic plants in the sections of rivers experiencing and not experiencing the impact of the storm system, the authors analyzed the effectiveness of using some statistical methods for summarizing data and their interpretation. The main task was to find the methods of analysis that would make it possible to interpret the situation as deeply as possible from a biological point of view.

## Materials & Methods

### Study site: Psel and Bystritsa Rivers

**Figure 1:**
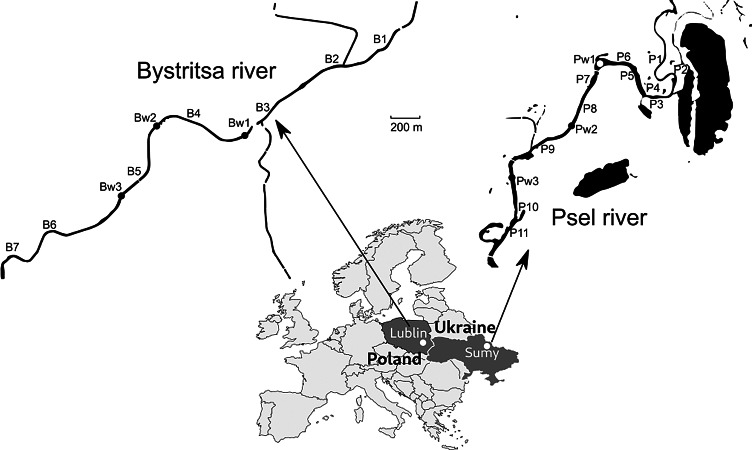
Map of the study area with sampling points.

Samples were collected on the Psel River, a left tributary of the Dnieper River (Black Sea basin) and the Bystritsa River, which flows from the left bank into the Wieprz River, a right tributary of the Vistula River-Baltic Sea basin (Fig. 1).

Both rivers are of the plain type, localized on the same geographical parallel, which suggests similar hydrological and climatic conditions. The rivers are more than 1,000 km apart and belong to different basins, which makes them independent research objects. The rivers were surveyed in sections flowing through the cities of Sumy (Ukraine) and Lublin (Poland) with approximately the same population. The studied sections on both rivers are straightened, deepened, the banks are partially turned into dams. In the studied areas, runoff from urban stormwater systems flows into the rivers. Both rivers are under the influence of channel reservoirs—a typical factor of disturbance of the natural hydrological regime of watercourses. The section on Psel river (Sumy) is located above the dam, and the section of the Bystritsa (Lublin) directly after the reservoir. This determined a significant difference in the flow velocity on the studied river sections. In the Psel River, the average flow velocity did not exceed 0.2 m/s, and in the Bystritsa River the flow velocity was in the range of 0.6–1.2 m/s. The low flow velocity in the Psel was determined by the dam located below, and in the Bystritsa high, not typical for plain rivers flow velocity was determined by water discharge from the dam and straightened canalized channel.

### Sampling method

Macrophytes are a convenient object of study, but their quantitative assessment creates difficulties and does not have standard generally accepted methods and approaches. An important factor in choosing the survey length is the total length of the river or body of water that will give a representative sample. The duration of the survey is directly related to the spatial frequency of the surveys (number of surveys per water body on a given date), since the length of the survey will generally decrease as the frequency of surveys increases (Directive 2000/60/EC, 2000). The most commonly used reach length is 100 m, described by Holmes (1991). Often 100-m is simply deemed a practical size of reach to survey. For example, national environmental agencies use a length of 500 m as recommended in the Guide to Common River Monitoring Standards (JNCC, 2005). A simple takeaway from the range of approaches available is that one size cannot fit all purposes. (Pentecost, Wilby & Pitt, 2009). In our studies, we proceeded from the fact that stormwater wobbling is most pronounced 10–20 m below the runoff. For this reason, we studied sections of 10 m. Below we are described in detail the methodology which we used.

Data on the projective cover of 20 aquatic plant species were used for the analysis. In total, data from 24 stations were analyzed, six of which were located at the discharge sites of stormwater systems. The material was collected both in places where stormwater systems flowed in (before and after the flow), and in areas where there was no influence of stormwater systems.

**Figure 2:**
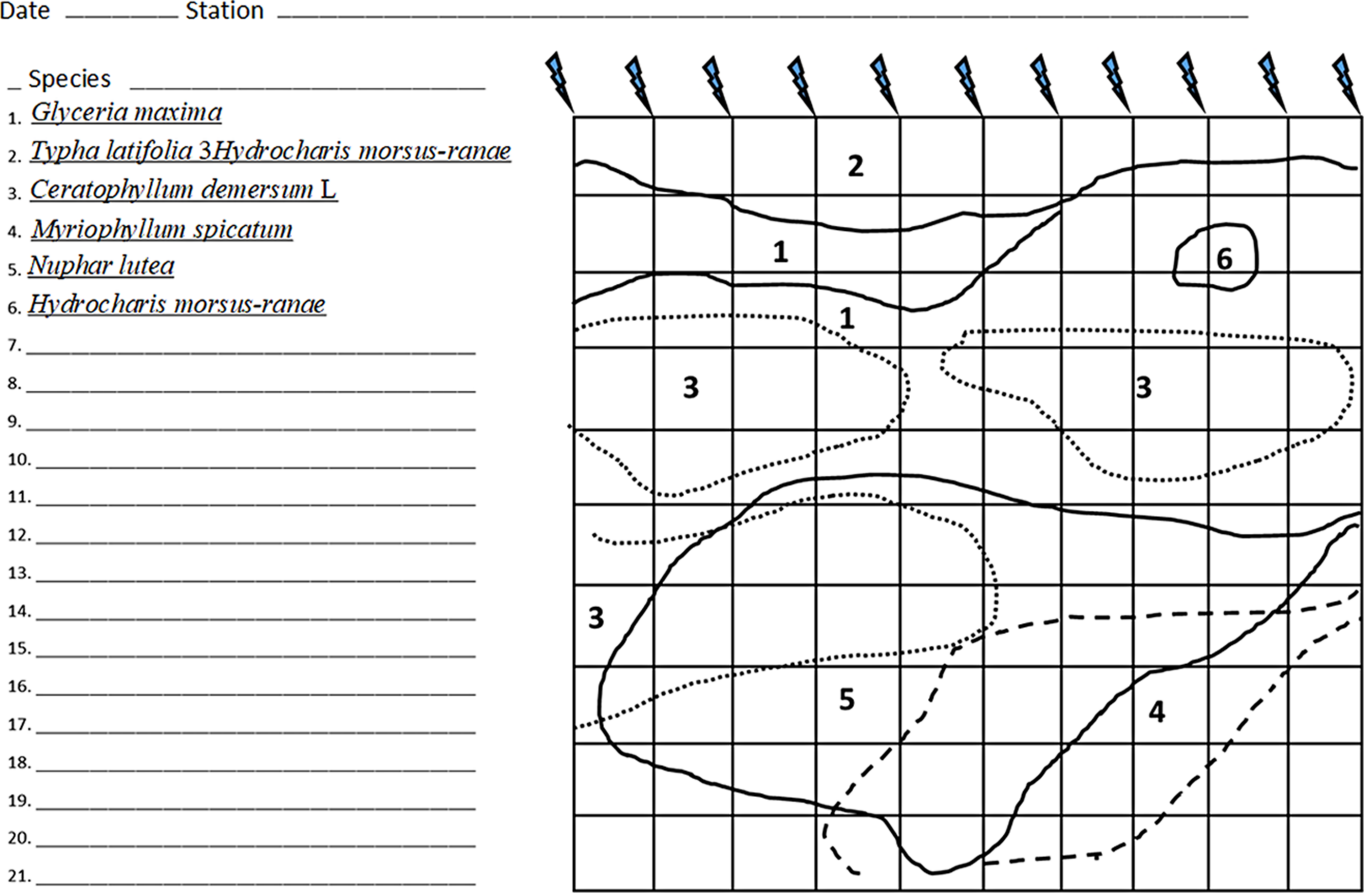
An example of a template with applied localization of macrophytes on a plot of 100 m^2^. Dashed lines indicate submerged macrophytes. - peg.

To determine the projective cover of macrophyte species at each station, the following methodology was used. The pattern of macrophyte patches was established by measuring their boundaries along transects spaced one meter apart using a tape measure. The results of the measurements were plotted on prepared templates (Fig. 2). Connecting the points on the template gives a fairly accurate macrophyte overgrowth pattern, allowing the proportion of overgrowth by species to be assessed. An area of 100 m^2^ was measured at each station (if the river width allowed).

The layout of macrophytes was plotted on the template. In the case of macrophytes with floating leaves, such as the yellow water-lily or waterlilies, the spot occupied by leaves was marked and the number of leaves in the spot was counted. We measured the area of 11 leaves to obtain the average area per leaf of a given species (of plant). To determine the projective area of the species, the average area was multiplied by the number of leaves within the study area. Separately, the coverage area of underwater macrophytes was estimated by plotting the configuration of their spots on a template. Single plants and small patches of vegetation were evaluated using a special floating frame divided by a wire mesh into squares of 1 dm^2^.

Coverage may have exceeded 100%, given the fact that plants with floating leaves constituted the second (upper) stratum and those submerged underneath constituted the first (lower) stratum.

The scientific plant names have been used according to Tutin et al. (1968–1980) and Dobrochaeva, Kotov & Prokudin Yu (1987).

### Statistical analyses

Ecological studies often use binary data (presence/absence) of species. Results based on abundance and presence/absence data often differ. Although we had abundance data we decided to consider the results of applying Jaccard distances to the converted to binary data. The data were processed using R version 4.0.2 (R Core Team, 2020). Hierarchical clustering was performed with hclust (function ward.D2) from core R package stats. The key to interpreting a results of hierarchical cluster analysis is to look at the point at which any given pair of cards “join together” in the tree diagram. Cards that join together sooner are more similar to each other than those that join together later (Albert & Tullis, 2008). Two types of *p*-values (AU and BP) for the nodes of hierarchical clustering dendrograms were calculated with R package pvclust. According to the package documentation, “AU *p*-value, which is computed by multiscale bootstrap resampling, is a better approximation to unbiased *p*-value than BP value computed by means of normal bootstrap resampling. Clusters with AU larger than 95% are highlighted by rectangles, which are strongly supported by data” (Suzuki, Terada & Shimodaira, 2019). Non-metric multidimensional scaling (NMDS) was performed with the metaMDS function from vegan (Oksanen et al., 2019). The NMDS method (Kruskal, 1964; Borg & Groenen, 2005) made it possible to obtain sets of points distributed in a 2-dimensional space in such a way that in the analyzed cases, the similar points were closest to each other, while different ones were spread far apart (Borg & Lingoes, 1987). Plots were produced using the ggplot2 (Wickham, 2016) and ggrepel packages (Slowikowski, 2020).

The Shannon–Wiener and Simpson’s indices were used to compare stations. Ordination in reduced space is often used in ecological studies so the authors considered results of nonmetric multidimensional scaling (NMDS) with different distance measures: Jaccard, Kulchinsky and Manhattan. In addition, the data were either preliminarily Hellinger transformed or not.

## Results

### Species composition

The species composition of macrophytes and average values of projective cover at stations both experiencing and not experiencing the influence of the stormwater system in the Bystritsa and Psel rivers are shown in Table 1.

### Projective cover

The projective cover of dominant species in the points affected by runoff on the Psel River reaches from 50% to 105%. At the same time, the share of individual dominant species varies from 15% to 35%. The total projective cover at these stations ranged from 85 to 120%.

Establishing the projective coverage of macrophytes is described in the ‘Materials and Methods’ section.

Projective cover of dominant species on the Psel River in areas not affected by runoff from stormwater systems ranged from 15% to 70%, with total cover remaining in the range of 39% to 72%. Participation of individual dominant species in these sites ranged from 10% to 30%.

The total projective cover in the Bystritsa River, which in the studied area is much narrower than the Psel River, ranged from 100% to 160% at stations affected by stormwater, while the projective cover of dominant species at these sites ranged from 66% to 117%.

**Table 1:** Macrophytes diversity and dominance (%) in the studied sections of the Psel and Bystritsa Rivers.

Macrophytes (arrange alphabetically)	Code	Psel River		Bystritsa River	
		Non-stop discharge station	Storm discharge stations	Non-stop discharge station	Storm discharge stations
*Acorus calamus* L.	Acocal	0.27 ± 0.26	–	–	–
*Ceratophyllum demersum* L.	Cerdem	13.3 ± 6.9	**20 ± 11.8**	6.6 ± 3.4	**18.5 ± 8.4**
*Glyceria maxima* Holmb.	Glymax	9.0 ± 8.1	0.67 ± 1.03	17.0 ± 6.71	3.67 ± 2.06
*Nuphar lutea* (L.) Sm.	Nuphlut	12.0 ± 6.8	**20 ± 4.5**	–	**–**
*Hydrocharis morsus-ranae* L.	Hydmors	0.7 ± 0.5	1.8 ± 1.7	0.64 ± 0.34	2.86 ± 1.45
*Lemna minor* L.	Lemmin	3.2 ± 4. 4	6.0 ± 4.7	1.57 ± 1.58	5.74 ± 2.72
*Myriophyllum spicatum* L.	Myrspic	0.4 ± 0.7	**20.33 ± 4.4**	1.71 ± 1.03	–
*Oenanthe aquatica* (L.) Poir.	Oenaqu	–	–	0.86 ± 0.67	–
*Persicaria amphibia* (L.) Delarbre	Peramph	0.5 ± 0.7	–	1.29 ± 0.79	–
*Phragmites australis* (Cav.) Trin. ex Steud.	Phraus	1.8 ± 3.0	–	10.29 ± 2.5	0.64 ± 0.34
*Potamogeton crispus* L.	Potcris	–	–	3.71 ± 1.57	7.97 ± 7.77
*Potamogeton natans* L.	Potnat	–	–	1.29 ± 1.16	4.36 ± 2.08
*Potamogeton perfoliatus* L.	Potperf	–	–	2.14 ± 2.29	–
*Sagittaria sagittifolia* L.	Sagsag	6.0 ± 5.5	2.7 ± 2.25	14.71 ± 13.32	1.54 ± 0.7
*Sparganium erectum* L.	Sparerect	0.3 ± 0.6	5.0 ± 1.18	1.0 ± 0.82	6.94 ± 3.48
*Sparganium emersum* Rehman	Sparemer	–	0.7 ± 1.0	21.43 ± 7.04	0.98 ± 0.64
*Spirodela polyrrhiza* (L.) Schleid.	Spipol	1.0 ± 1.4	1.7 ± 1.4	0.71 ± 0.67	5.61 ± 2.65
*Stuckenia pectinata* (L.) Böerner	Spotpect	–	–	1.71 ± 1.43	**20.89 ± 9.66**
*Typha angustifolia* L.	Typhang	4.8 ± 5.5	–	10.43 ± 6.31	–
*Typha latifolia* L.	Typhlat	0.5 ± 1.0	**20.0 ± 4.5**	5.14 ± 3.1	**16.32 ± 7.59**

**Notes.**

– absence of species.

bold numbers indicate the dominant species.

In sections of the Bystritsa River not directly influenced by runoff, the total projective cover reached a maximum of 125% and a minimum of 95%. At the same time, on these sites dominant species could occupy from 31% to 73%. In areas influenced by runoff, dominant species occupied 66% to 117%, with total cover ranging from 107% to 160%.

### Similarity

The similarity of species composition of macrophytes at stations influenced by stormwater and stations not influenced by them in the Psel River was 80% according to the Sørensen index, and in the Bystritsa River—85%. At the stations in the Psel River influenced by runoff, 11 macrophyte species were found, while 14 species were found at stations not influenced by runoff; at the Bystritsa River these numbers were 13 and 18 macrophyte species, respectively.

Table 2 presents species richness and biodiversity indices for non-stop discharge station and storm discharge stations.

**Table 2:** Species richness and biodiversity indices in studied sections of Psel and Bystritsa Rivers.

Biodiversity measures	Psel River		Bystritsa River	
	Non-stop discharge station	Storm discharge stations	Non-stop discharge station	Storm discharge stations
Mean number of species	8.27 ± 0.973	9.3 3±0.882	16.9 ± 0.553	14.0 ± 0
Species richness	14	11	18	13
Shannon-Weiner index	1.66 ± 0.131	1.79 ± 0.130	2.30 ± 0.0371	2.18 ± 0.0642
Simpson’s index	0.769 ± 0.0277	0.803 ± 0.0292	0.867 ± 0.00652	0.863 ± 0.0143

### Indicative groups

**Figure 3:**
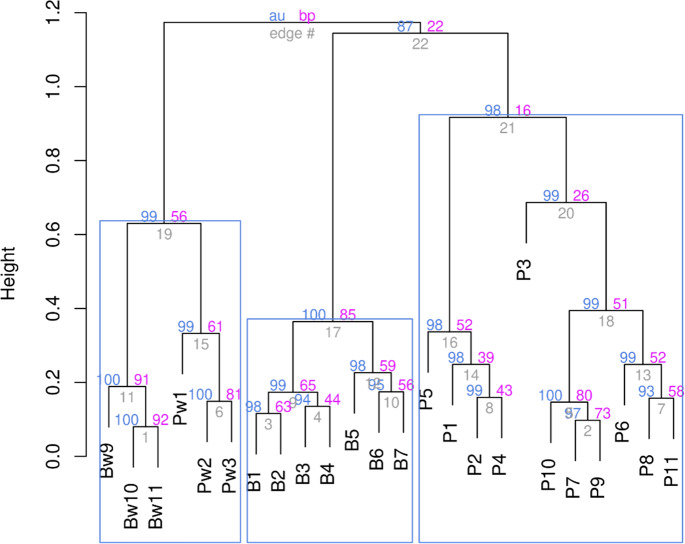
Ward’s hierarchical clustering of stations based on the qualitative composition and quantitative development of aquatic plants with Hellinger transformed data and the Kulczynsky distances.

The proximity of all studied stations to each other was assessed using hierarchical clustering according to the Ward’s method. The data were previously Hellinger-transformed; for clustering, the Kulczynsky distance matrix was used (Fig. 3).

The hierarchical clustering showed that all stations with high reliability—as evidenced by the *p*-values based on multiscale bootstrap resampling (see Fig. 3)—split into three clusters. The stations of both rivers affected by stormwater runoff form a separate cluster. At the same time, stations not affected by runoff are distributed in the remaining two clusters according to the rivers. The fact that all stations of both rivers affected by stormwater systems were combined into one cluster suggests that the impact of runoff is the decisive factor and, according to the cluster analysis, the impact of the stormwater system was more significant than river affiliation. Indeed, if the influence of stormwater is not decisive, one should expect the stations to be divided into two groups, according to their localisation on different rivers. However, stormwater runoff was found to even out the differences between rivers, resulting in similar conditions, as reflected in the cluster analysis.

**Figure 4:**
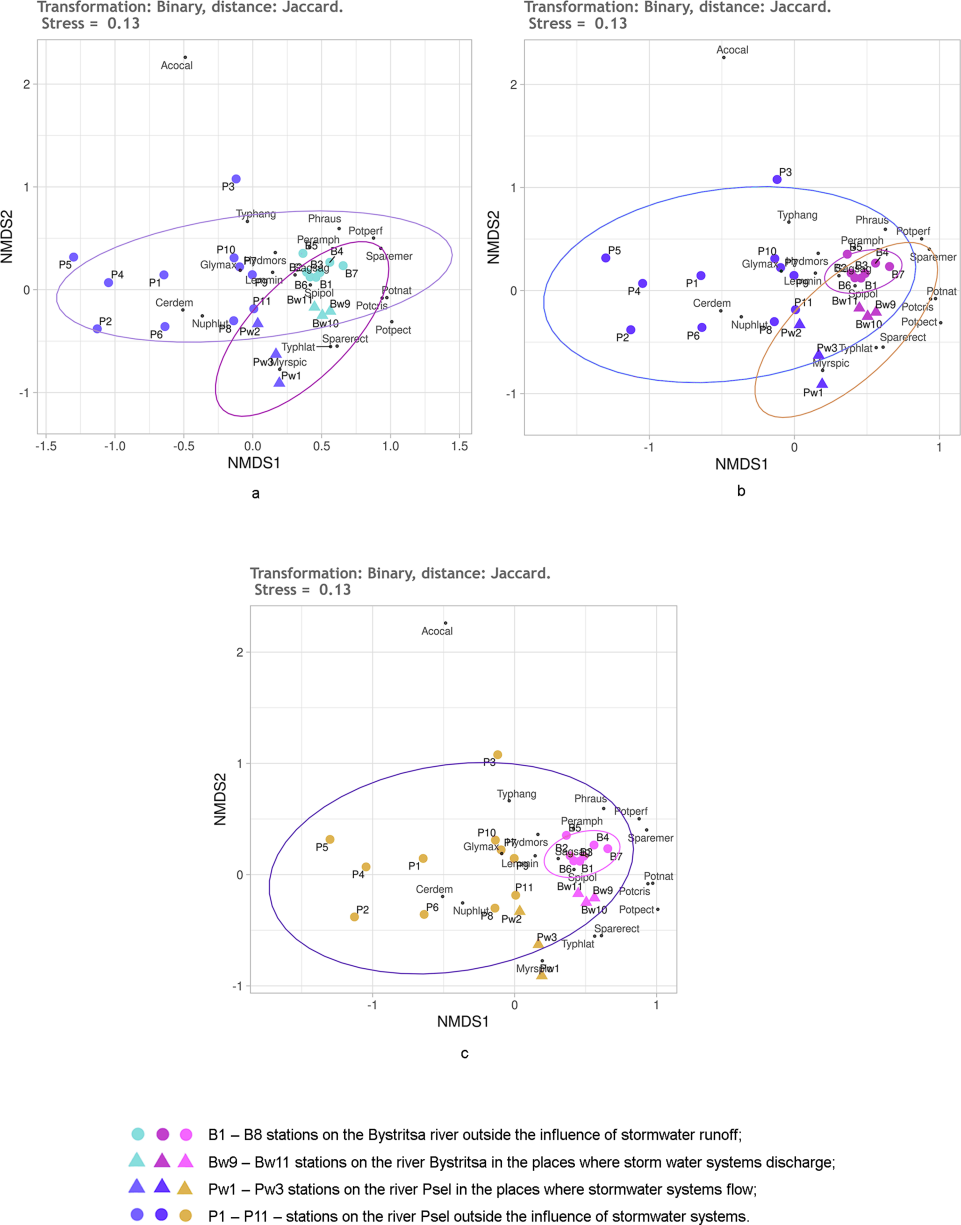
NMDS of stations on the Psel and Bystritsa rivers affected by runoff from stormwater systems using the Jaccard distance matrix; (A) with the allocation of two groups; (B) with the allocation of three groups; (C) with four groups; (B1)–(B8) stations on the B.

Figure 4 shows the NMDS results with Jaccard distances. No matter how many groups are selected—two, three or four—95% confidence ellipses overlap more or less (Fig. 4). When distinguishing two groups (Fig. 4A): stations of both rivers without the influence of the storm system and stations of both rivers in the area of runoff from the storm system. All stations of the Bystritsa river fall within the confidence ellipse for a group of stations in the area of runoffs. When divided into three groups: the stations unaffected by stormwater runoff on the river Psel, stations on the river Bystritsa and the stations of both rivers in the runoffs regions show that the NMDS of the matrix with Jaccard distances does not provide an opportunity to separate these groups.

Even more clearly, the impossibility of distinguishing the groups of stations based on Jaccard distances is seen in Fig. 2C when setting four groups: two for the stations of each river under the influence of storm system and without. Without exception, all the studied stations: on the river Psel, and river Bystritsa fall into a single 95% confidence ellipse for the Psel River group (Fig. 4C). Thus, the NMDS analysis based on qualitative data demonstrates only the commonality of the studied objects and does not allow speaking about reliable differences between them.

All further results were obtained using the quantitative data on the participation of individual species in the projective cover of the water body at the study stations. The division of stations into groups was the same as described above, so further on the authors will simply consider the two, three or four groups without detailing.

**Figure 5:**
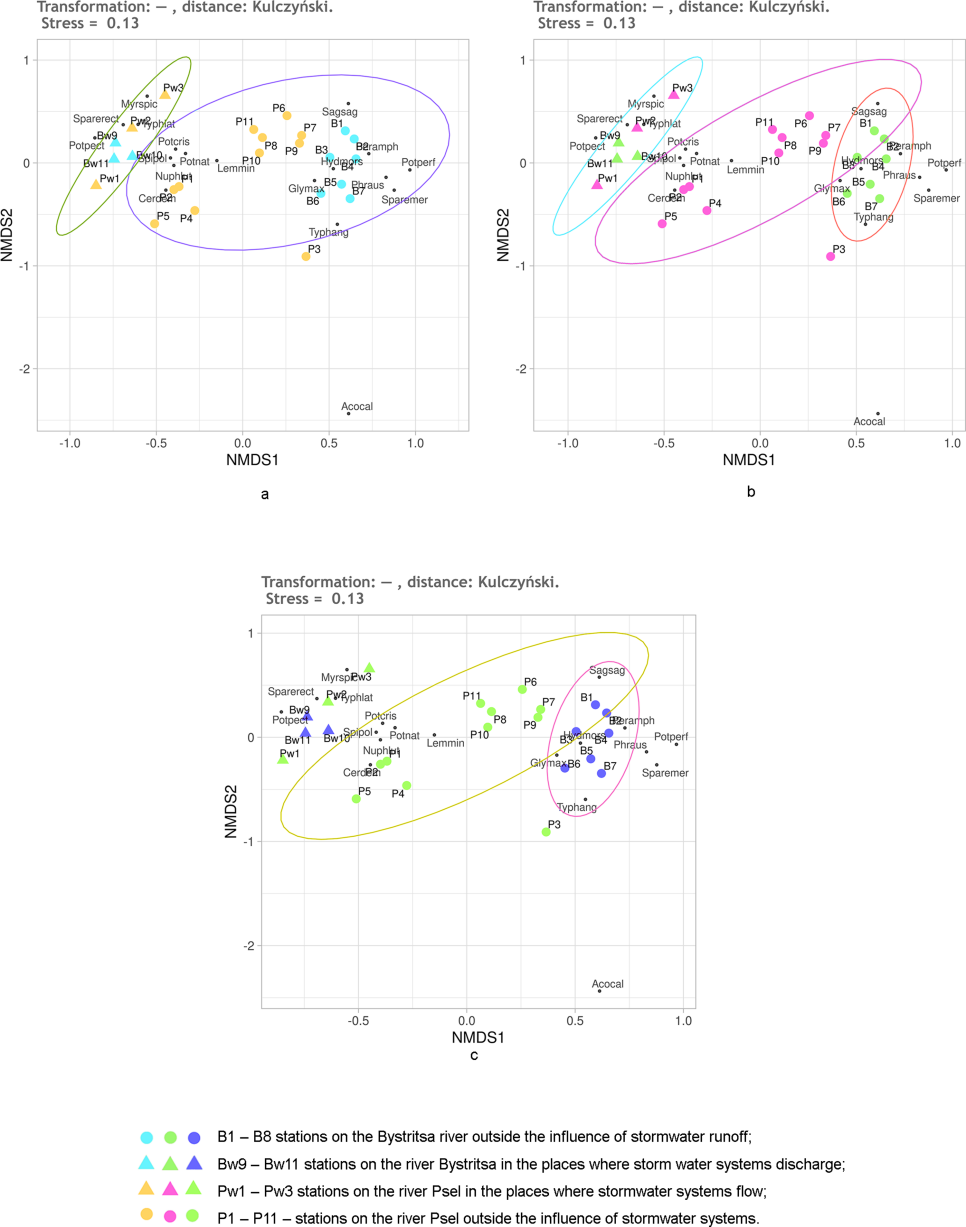
NMDS of stations on the Psel and Bystritsa rivers affected by runoff from stormwater systems using untransformed data and Kulczynski distance matrix; (A) with the allocation of two groups; (B) with the allocation of three groups; (C) with four groups. The designations are the same as in Fig. 2.

First, it was checked how the untransformed data work using the Kulczynski distance. The NMDS results are shown in Fig. 5. Regardless of the preassigned number of groups, the results are quite similar. When two or three groups are assigned, only the stations of both rivers under the influence of stormwater and stations on both rivers without the influence of stormwater are significantly different. An attempt to distinguish four groups (Fig. 5C) is also unsuccessful, since the points of stations of both rivers, which are under the influence of runoffs, lie mixed up on the NMDS plain and do not form two groups. Confidence ellipses for the stations without stormwater effect overlap. Thus, the NMDS, on the basis of untransformed data with the Kulczynski distances, prompts the conclusion that the objects of two types are being dealt with: rivers without the influence of stormwater systems and rivers in the area where stormwater runoff flows. This gives grounds to assert that the influence of runoffs is significant, leading to the destruction of the individuality of the river.

As it can be seen, using the Kulchinsky distances on the raw data deepened the understanding, compared to using the Jaccard distances.

**Figure 6:**
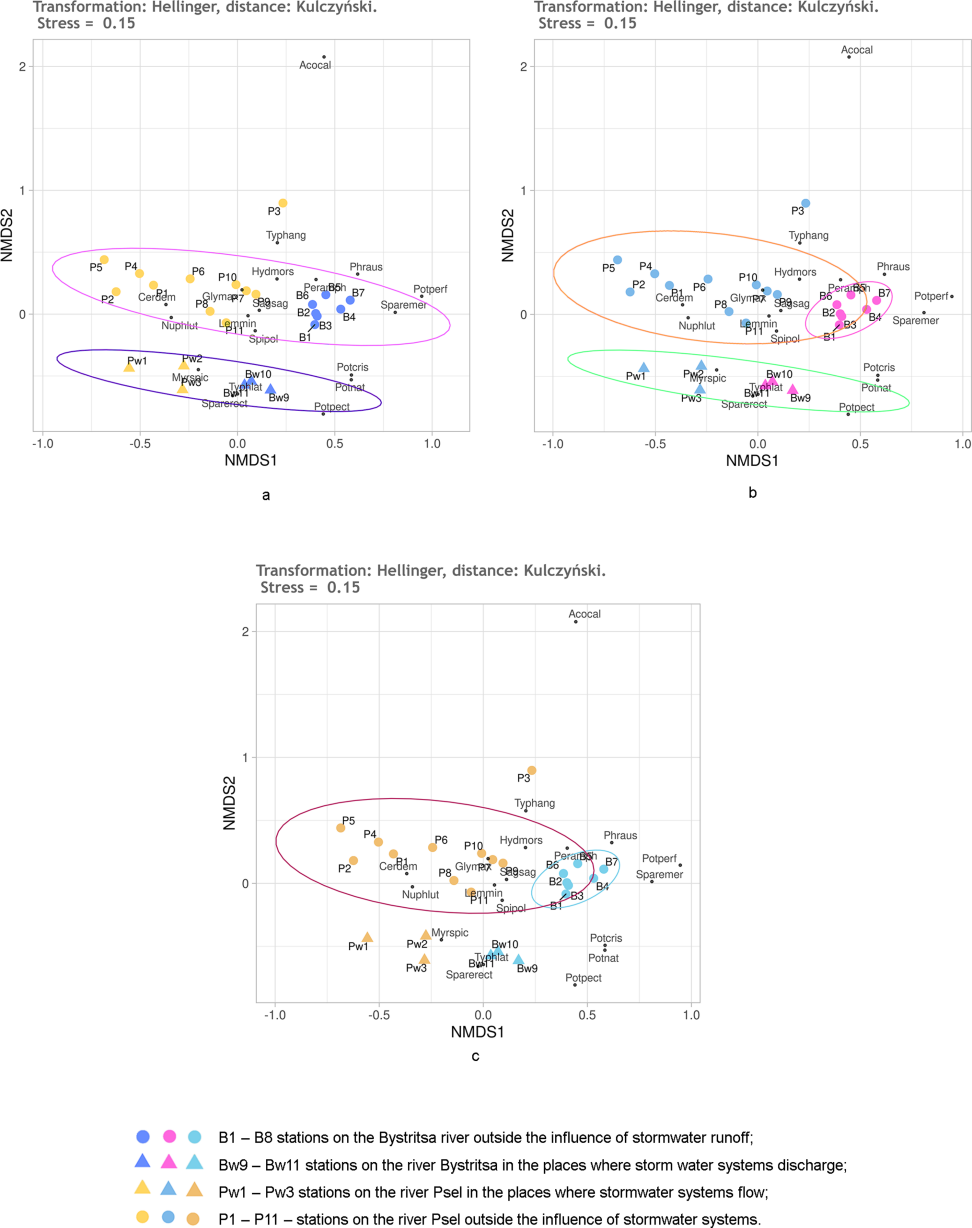
NMDS of the stations on the Psel and Bystritsa rivers affected by the runoff from stormwater systems using Hellinger transformed data and Kulczynski distance matrix; (A) with the allocation of two groups; (B) with the allocation of three groups; (C) with four groups. The designations are the same as in Fig. 4.

It is known that Hellinger transformation of a data (Legendre & Gallagher, 2001) often contributes to better ordination results. Therefore, it was used before calculating the Kulczynski distances (Fig. 6). Here, the reliability of different groups is the same as without transformation, but the relative position of stations on the diagram is different. The position of the stations under the influence of stormwater system is especially different (Fig. 5). Without transformation, these stations of both rivers are mixed up and positioned much closer to the stations of the Psel River than the Bystritsa River. After transformation according to Helenger, the unaffected stations of both rivers are more close with their own river’s stations affected by stormwater runoffs. This allows refining the interpretation of the results. It can be stated that runoff locally transforms the composition and share of different aquatic vegetation species in the projective cover of each river, but some characteristic features of each hydro-ecosystem remain. It should be noted that when using the Jaccard distance, the stations affected by runoff did not stand out as a separate group, but in all cases had a distinct tendency to be near the stations of the corresponding river (Fig. 4).

**Figure 7:**
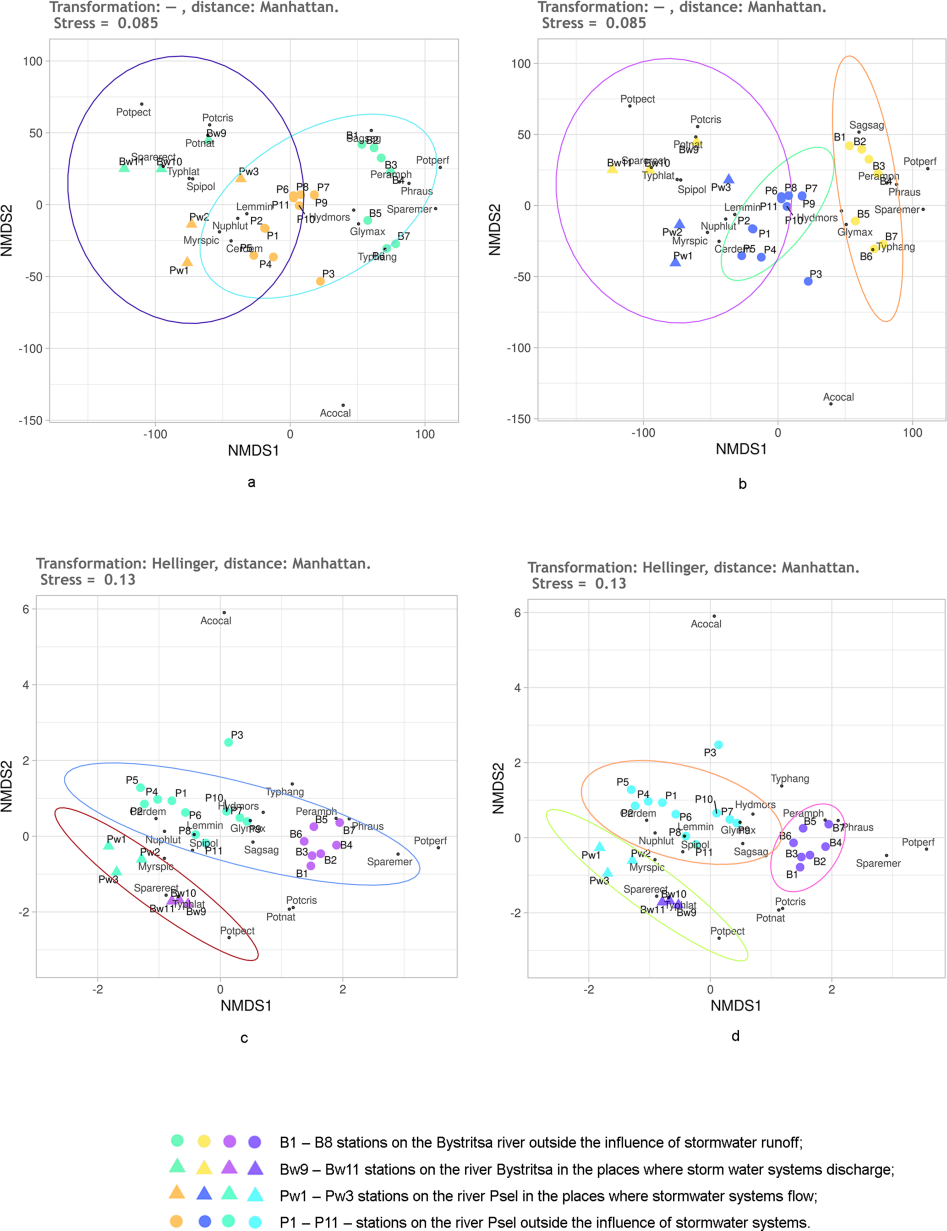
NMDS of stations on the Psel and Bystritsa rivers affected by runoff from stormwater systems using untransformed data (A, B) and Hellinger transformed data (C, D).

The effect of Helenger transformation on NMDS results can also be illustrated by using Manhattan distance. Thus, without the Helenger transformation, the predefined groups on the plot overlap and do not provide a basis for a confident interpretation (Figs. 7A, 7B). After transformation according to Helenger, in both cases, a group is distinguished that unites stations affected by the runoff, and the unaffected stations from different rivers are also quite confidently separated. This coincides with the previously obtained subdivision of stations by the clustering method.

**Figure 8:**
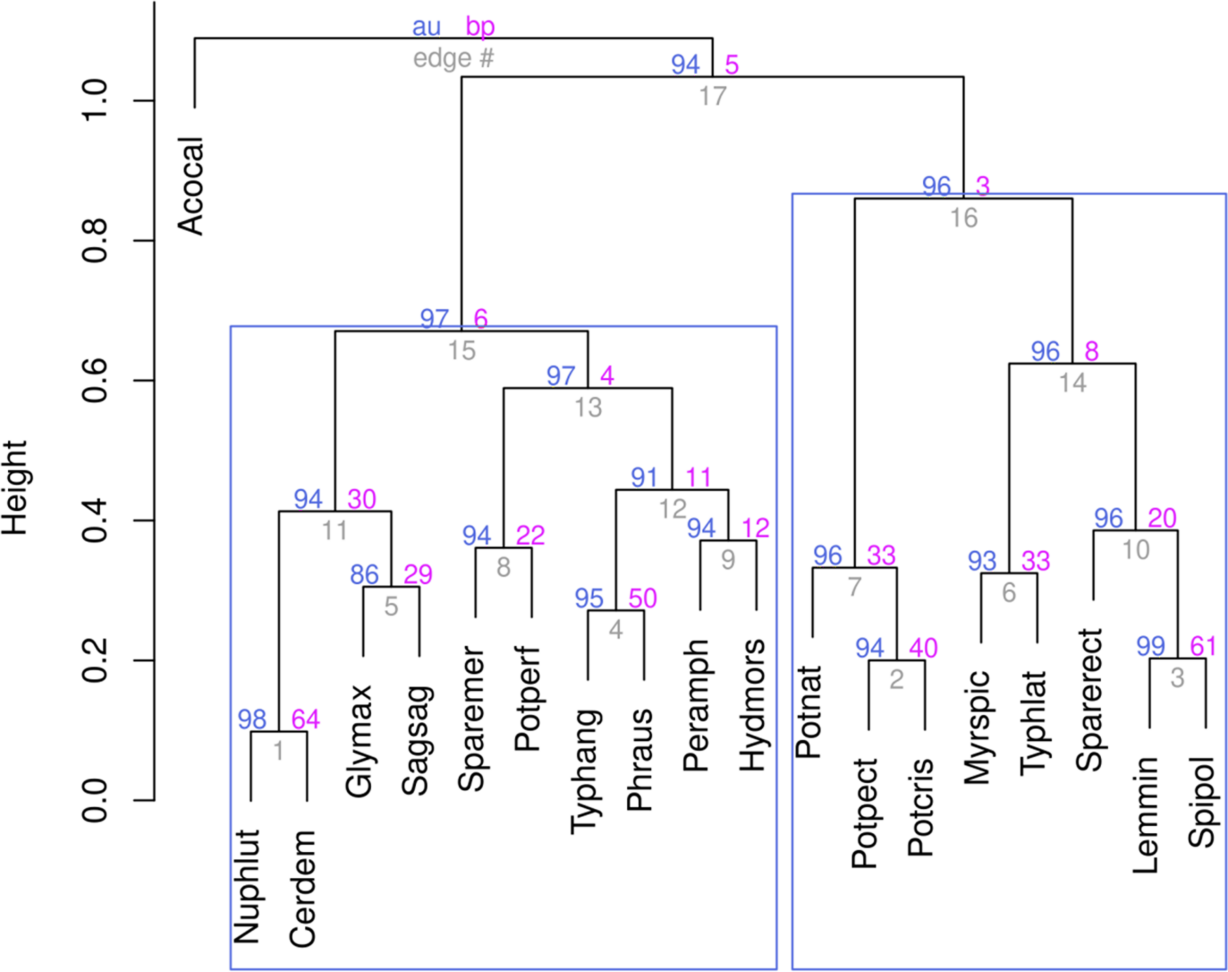
Ward’s hierarchical clustering of aquatic plant species. The data were Hellinger transformed, distances of Kulczynsky.

Analysis of the positions of stations on the graphs and associated plant species on the basis of hirearchical clustering (Fig. 8, right cluster) and NMDS results makes it possible to identify a group of species associated with the influence of runoff from stormwater systems. In the area of wastewater discharge from the stormwater system on the Psel River the dominated species were: *Nuphar lutea*, *Ceratophyllum demersum*, *Myriophyllum spicatum*, *Typha latifolia*, and on the Bystritsa River were: *Glyceria maxima*, *Typha latifolia*, *Stuckenia pectinata* and *Potamogeton crispus*.

## Discussion

The storm runoffs affect the biocenoses of rivers. The pollutants that come with stormwater can be buried in bottom sediments or accumulate in the tissues of aquatic biota (Gołdyn et al., 2018; Barałkiewicz et al., 2014). Many studies have shown that macrophytes are able to significantly reduce the concentration of harmful substances (Ladislas et al., 2012) and be indicators of the quality of the environment (Kohler & Schneider, 2003; Maissour & Benamar, 2019). The work of (Barałkiewicz et al., 2014) showed that a reservoir located in the riverbed can contribute to the elimination of pollutans from stormwaters, despite the very short residence time of water in it. The macrophyte thickets in the reservoir and the biota associated with them played the main role in improving the water quality. As recent studies show, as a rule, there is no unambiguous relationship between the species composition of macrophytes and the ecological conditions in rivers. Most likely, this is due to the fact that stormwater drains differ significantly from ordinary point sources of pollution: effluents from industrial or agricultural enterprises, treatment facilities, *etc*. The said effluents have fairly stable volumes and composition, as well as the level of their treatment. Storm runoffs are much less constant, they depend on the weather conditions, area and features of the use of the territory from which they are collected. All this makes their composition and volume very relatively predictable.

As a rule, in environmental studies, it is important to the degree of disturbance of the ecosystem or the intensity and significance of the influence of a local factor or factors assess as objectively as possible; in our case, this corresponds to the effect of the runoff from stormwater systems on rivers. It is obvious that the assessment of the influence of a factor made on the basis of various indicators may differ, opening up the possibility for different interpretations. The use of various statistical methods based on one material, will assess their effectiveness and objectivity. At the same time, the use of several methods or indicators allows one to expand and deepen the interpretation of the results.

In general, on the basis of the analysis performed, it can be argued that the use of the NMDS method provides good insight into the structural rearrangements in marophyte communities affected by runoff from stormwater systems.

The local influence of rainfall runoff is visually well noticeable and manifests itself in an increase in the area occupied by macrophytes. In the perfomed studies, a significant increase in the number of macrophytes was also observed at the place where the runoff flows and downstream. However, differences in the scale of rivers and, especially, in the speed of the current (Grinberga, 2011), made significant adjustments.

In both rivers, the species composition of macrophytes was quite similar. Hovewer in Bystritsa, one species found in Psel was not noted: *Acorus calamus L*. At the same time, species of the genus *Potamogeton* were absent in the studied stations of Psel: *P. crispus, P. natans, P. perfoliatus* us and *Stuckenia pectinata*.

Like most members of the genus *Potamogeton*, these four species occur in both enclosed bodies of water and slow-flowing rivers. However, specific conditions can either favour or hinder the development of the species. For example, *Pot. crispus* thrives in early summer when temperatures are relatively low. In the Psel river the slow running dark water gets very warm already from the beginning of spring. Species of *P. natans* and *P. perfoliatus* are inhibited in water bodies with increased anthropogenic pollution, which is the case in the study area. However, the pollution-tolerant *Stuckenia pectinata* was also absent. It could be assumed that this was due to the conditions of the river section. The river channel is straightened and deepened here and shallow waters are practically absent. Bed sediments are represented by mobile suspended silt, in some places over 1 m. This nature of the substrate prevents the rooting of the *Potamogeton* species which does not have a well-developed root system. Nevertheless the similarity of the species composition of macrophytes according to the Sørensen index between the two river is 82%. In the places where stormwater flows in, the species composition in both rivers was also quite similar, judging by the values of the Sørensen index: 80%. Obviously, a significant difference in the current velocity made adjustments to the composition of the dominant species at the places where stormwater flows. The similarity between the composition of the dominant species of macrophytes in the places of stormwater inflow according to the Sørensen index is 67% complex of macrophyte species is apparently also caused by the difference in the flow rate in the studied river sections. This corresponds to the research results published by Grinberga (2011). As already mentioned, in Psel there were no representatives of the genus *genera Potamogeton* and *Stuckenia*, which are included in the complex of dominant species at the places where stormwater flows into the Bystritsa river.

The Bystritsa River within the city of Lublin is represented by a section below the reservoir. The river bed is canalized; the speed of the current in some places reaches 1 m/s. At all the studied points, where storm runoffs occurred, the area of overgrowth with macrophytes was increased, the “spots” of macrophytes were limited to 20–40 m downstream stormwater runoff.

On the studied section of the Psel River in Sumy, the channel is also canalized, but the section is located upstream the dam of the hydroelectric power station, which leads to a decrease in the flow rate to 0.1−0.3 m per second. Accordingly, the overgrowth with macrophytes at the place of inflow of the drains was observed here at a distance of 100–150 m downstream of the places of confluence of storm water systems.

The differences in the reaction of macrophytes on both rivers were significant, but as the conducted studies showed, the influence of storm systems has much in common. In general, on the basis of the statistical analysis carried out, it can be argued that the features of the composition of the flora of each river are important, but the influence of storm runoffs largely neutralizes the specificity of the rivers.

Thus, macrophytes, by accumulating the substances introduced by wastewater, play an important role as a biofilter, limiting the spread of the negative impact of stormwater systems on the river.

It should be noted that the runoff of most stormwater systems is located in close proximity to the coast, which determines the presence of a zone of influence in the form of macrophyte spots. Probably, if a relatively short channel is formed between the point of inflow of the runoff and the water body, then the main load from the runoff will be taken by the thickets of macrophytes within this channel. Periodic removal of plants, for example, with accumulated heavy metals, can significantly reduce the load on the river.

## Conclusions

The use of clustering and NMDS gives grounds to assert that:

(1) The runoff from stormwater system is definitely locally changing the situation for aquatic vegetation in the rivers.
(2) The runoffs from stormwater drainage make sections of different rivers quite similar to each other, significantly nullifying their features.This nullification of differences is mainly determined by the increase in biomass and projective cover in the area of runoff.
(3) The similarity of macrophyte species compositions between sites affected by storm water runoff and stations free from the influence of runoff on the Psel River was 80%, and on the Bystritsa River—85%.
(4) In the conditions of both rivers two species of macrophytes: *Ceratophyllum demersum*, *Typha latifolia* responded to the impact of runoff by increasing the area of overgrowth. In the conditions of Bystritrzyca, pondweed, *Stuckenia pectinata* (*Potamogeton pectinatus*) and in the Psel—*Myriophyllum spicatum* and *Nuphar lutea*—are added to this complex of species. The response of these species makes them indicative of the impact of stormwater systems on the structure of macrophyte communities.
(5) The use of the NMDS method provides good insight into the structural rearrangements in macrophyte communities affected by runoff from stormwater systems.

##  Supplemental Information

10.7717/peerj.15248/supp-1 Supplemental Information 1 Numbers of macrophyte communities at individual stations on the Psel and Bystritsa rivers

## Additional Information and Declarations

### Data Availability

The following information was supplied regarding data availability: The raw data is available in the Supplementary File.
